# Research Priorities for Drivers of Food Choice for Food System Transformation in South Asia: Proceedings of a Collaborative Workshop

**DOI:** 10.1016/j.cdnut.2025.104582

**Published:** 2025-03-10

**Authors:** Christine E Blake, Sunny S Kim, Edward A Frongillo, Purnima Menon

**Affiliations:** 1 University of South Carolina, Arnold School of Public Health, Columbia, SC, United States; 2 International Food Policy Research Institute, Washington, DC, USA

**Keywords:** food choice, food environment, food systems, sustainable healthy diets, South Asia

## Abstract

Agrifood systems in South Asia are highly productive, but substantial challenges including poverty, climate change, and environmental degradation complicate progress toward achieving sustainable healthy diets for all. The dynamics of food systems and the consequence of their rapid transformation for food choice behaviors that contribute to healthy and unhealthy diets are not well understood. Food choice is defined as a decision-making process through which individuals and households consider, acquire, prepare, distribute, and consume foods and beverages. Understanding drivers of food choice (DFC) is important for achieving sustainable healthy diets, but evidence is lacking. This article outlines collectively derived priorities for future research on DFC in South Asia. A collaborative workshop was convened in March 2023 in Dhaka, Bangladesh, with experts from the region. The workshop emphasized the application of a science of food choice framework to guide identification of priorities for research on DFC in South Asia. Priorities were derived through an interdisciplinary collaborative process to clarify what is known and not known about DFC in the context of Food Systems Transformation in the region with emphasis on a continuum of food choice behaviors (production, acquisition, preparation, distribution, and consumption). Workshop participants identified the following 3 main priorities for future research on DFC that address knowledge gaps that emerged from discussions: *1*) intrahousehold dynamics and behaviors, *2*) adolescent food choice, and *3*) market and food acquisition linkages. Specific research needs to emphasize the importance of multigenerational data, food allocation, perceptions on food safety, adolescent food choice behaviors, and the need for longitudinal data on linkages between market availability and food choice behaviors. Building a body of evidence on DFC and tools for monitoring and assessing food choice behaviors is essential for designing effective policies and programs that allow all individuals to have healthy and sustainable diets in South Asia.

## Introduction

Agrifood systems in South Asia are highly productive with the capacity to feed the ∼25% of the world’s population residing in the region, but substantial challenges including poverty, climate change, and environmental degradation impede progress toward achieving sustainable healthy diets for all [[1], [2], [3], [4]]. Further, social, economic, and geographic inequities create barriers across the food system with ongoing food security challenges and increasing amounts of unhealthy food. Rates of undernutrition in South Asia as a share of the total population were steadily improving until 2018, but a complex set of intersecting social, environmental, political, and health challenges have stalled continued progress [5]. A quarter of the population in South Asia lives below the poverty line [[6], [7], [8]], and half of the population are unable to afford a healthy diet that meets the needs for health, well-being, and optimal growth and development [[9], [10], [11], [12]].

Food systems have been defined as the “sum of actors and interactions along the food value chain—from input supply and production of crops, livestock, fish, and other agricultural commodities to transportation, processing, retailing, wholesaling, and preparation of foods to consumption and disposal” [13]. Availability of food in markets is more closely aligned with consumer diets than home food production in South Asia [[14], [15], [16]], and low-income households in rural areas increasingly rely on food markets rather than own-production [17]. Along with persistent undernutrition, increasing trends of ultraprocessed food production and consumption across all countries and socioeconomic groups are an emerging challenge, especially among the poor [4]. Rising prevalence of overweight/obesity and noncommunicable diseases, rapidly changing food environments, and climate-driven disruptions necessitate solutions to these complex interrelated challenges across the food system [[18], [19], [20]]. The dynamics of food systems and the consequences of their rapid transformation are still poorly understood [21]. Addressing gaps in our understanding of food systems dynamics in the South Asia region requires investigating behavior at the nexus where households and individuals interact with their food environments.

Household and individual food choice behaviors play an integral role in all food systems. Food choice is defined as a decision-making process through which consumers consider, acquire, prepare, distribute, and consume foods and beverages [2]. Decisions that consumers make about what, how, and why to eat are constrained by what is available and accessible within the local food environment [22,23]. There are multiple drivers of food choice (DFC) (e.g., cost, taste, convenience, habit, and culture) at different levels of influence (e.g., personal, community, and market) [2,3,24,25]. Even in the most disadvantaged settings, consumers can and will exercise agency in their food choice behaviors to the extent possible, given their real and/or perceived constraints and limitations (e.g., cost, time to acquire or prepare, and facilities to prepare) and personal perspectives (e.g., cultural acceptability, taste preferences, and health needs) [26]. What, how, and why people eat the way they do must be understood within the biological, economic, cultural, social, and environmental context in which decisions are made [2,3]. Knowledge about DFC behaviors provides the necessary foundation for successful demand-driven research and programmatic agendas for food system transformation that seeks to alter behavior across the food system. Attention to and study of the DFC behaviors in South Asia has increased in recent years, but evidence to guide action is lacking [27]. A recent review of food choice research found only 20 studies on food choice for all of South Asia with most studies focusing on the role of sensory appeal and social interactions [26]. Work in this region has been fragmented, prompting calls for greater cross-country collaboration and knowledge sharing in this area. Sustaining recent progress and responding to new challenges to address malnutrition in all its forms in South Asia requires a clear and comprehensive understanding of DFC behaviors in context.

The Drivers of Food Choice Competitive Grants Program (DFC-CGP), launched in 2015, facilitated, synthesized, and disseminated research to provide a deep understanding of the DFC across different vulnerable population groups in different lifecycle stages in low-income and middle-income countries (LMICs) of South Asia and Sub-Saharan Africa [28]. The DFC-CGP yielded a high-quality portfolio of multidisciplinary research on the DFC [2]. The DFC-CGP also generated a science of food choice framework to guide the framing of existing knowledge and situating new evidence on DFC. Ultimately, this program has provided valuable insights for policymakers and global health specialists working to address the food and nutrition needs of vulnerable populations in LMICs [28] and fostered a vibrant community of practice working to better understand food choice in the context of Food Systems Transformation.

The One Consultative Group on International Agricultural Research (CGIAR) is a unified global research partnership dedicated to transforming food, land, and water systems through research and innovation to achieve sustainable, food-secure, climate-resilient communities. Action areas being addressed through collaborative networks include systems transformation, resilient agrifood systems, and genetic innovation. Actions by One CGIAR are part of ongoing global efforts by multiple organizations working on this topic. In 2022, One CGIAR launched several research initiatives to address challenges in these action areas [29], including Transforming Agrifood Systems in South Asia (TAFSSA), Fruits and Vegetables for Sustainable Healthy Diets, Resilient Cities, and Sustainable Healthy Diets through Food Systems Transformation, and Resilient Cities [30]. In particular, the TAFSSA initiative was developed to deliver a coordinated program of research and engagement across the food system to support equitable access to sustainable healthy diets; improve farmer livelihoods and resilience; and conserve land, air, and groundwater resources in the South Asia region [31]. Equitable access is conceptualized as ensuring fair access and equal opportunity accounting for differences in life circumstances. TAFSSA use multiple strategies that aim to propel evidence into impact through engagement with public and private partners. An essential component of TAFSSA is understanding the behavioral and structural determinants of sustainable healthy diets by studying dietary practices of food consumers, identifying DFC, and testing innovations to support consumption of sustainable healthy diets. Sustainable healthy diets are defined as “dietary patterns that promote all dimensions of individuals’ health and well-being; have low environmental pressure and impact; are accessible, affordable, safe and equitable; and are culturally acceptable” [32]. The TAFSSA initiative works with partners in 4 countries—Bangladesh, India, Nepal, and Pakistan—to address emerging challenges and to leverage emerging opportunities for the successful transformation of food systems.

This article identifies priorities to clarify what is known and not known about DFC in the context of food systems in South Asia and outline future research on food systems in South Asia derived through an interdisciplinary collaborative process, led by the TAFSSA team and the DFC-CGP team at the University of South Carolina, with emphasis on behaviors across the food system.

## Methods Workshop: Assessing DFC in South Asia

The TAFSSA team, in collaboration with the DFC team at the University of South Carolina, codesigned and convened a hybrid (in-person and online) workshop on methods for assessing DFC in Dhaka, Bangladesh, from 14 to 16 March 2023. The motivation for holding this workshop came from the recognition that evidence to guide action on food choice behaviors for Food Systems Transformation in South Asia is lacking or fragmented. This workshop aimed to identify ways to address the evidence gap and promote greater within and across country collaboration and knowledge sharing in this area.

The workshop applied a science of food choice framework and used learnings about research designs and methods from DFC syntheses of evidence to better understand the determinants of sustainable healthy diets and identify DFC in South Asia. The workshop was designed to provide participants with the knowledge needed to develop analytic protocols for assessment of food choice behaviors, drivers of these behaviors, and relationships of individual food choice behavior to food environments in the TAFSSA communities. Emphasis was placed on the need for innovation in studying DFC. The ongoing work in TAFSSA focus countries of Bangladesh, India, Nepal, and Pakistan was used to frame discussions the scope of the discussion extended beyond this single project. Participants collectively identified food choice behaviors and their drivers and the information and analyses needed for policy and program decision making in South Asia. The workshop sessions also encouraged the development of new collaborations with local partners and across CGIAR Initiatives working in the region. The design plans and materials for the TAFSSA local agrifood systems assessment survey, which was later conducted in February–April 2024 in 5 districts across Bangladesh, India, and Nepal, provided context for the workshop activities [33].

Workshop participants included researchers from several CGIAR centers, i.e., International Food Policy Research Institute, International Maize and Wheat Improvement Center, International Rice Research Institute, and International Water Management Institute involved in the One CGIAR initiatives of TAFSSA, Fruits and Vegetables for Sustainable Healthy Diets, Sustainable Healthy Diets through Food Systems Transformation, and Resilient Cities. The workshop participants were from numerous countries in South Asia, including India, Bangladesh, Nepal, Sri Lanka, Pakistan, and Indonesia. Researchers from universities and research centers were represented such as Aga Kahn University (Pakistan), Wayamba University (Sri Lanka), and SEAMEO RECFON University (Indonesia). Many participants were researchers and technical experts involved in food systems research across South Asia from academia, government institutions, national research organizations, and international and national nongovernmental organizations.

## Workshop Sessions

The workshop comprised 6 sessions, held over 3 d (Table 1). The agenda was developed to include presentations from the CGIAR food systems initiatives to share how they are approaching their research on DFC, as well as from other research groups to share experiences of field studies on DFC and on global measurement methods. The agenda was designed with breakout sessions to facilitate small group discussions. There were also focused crosscutting discussions about how to capture gender and equity considerations as part of the methods development.

**TABLE 1:** Summary of workshop session topics and activities

Session topics and speakers	Activities and outcomes
Session 1: Elaborating the science of food choice: understanding what, how, and why people eat the way they do	Introductory presentations to orient participants
Session 2: Understanding food choices Constructs and contexts Measurement experiences Introduction to the DFC assessment methods repository	Review of recent conceptual and methodological advances in food choice research in LMIC. Introduction of participants to the DFC Assessment Methods Repository (β)
Session 3: Identifying problems and needs for actionable evidence	Overview of TAFSSA country sites as contexts for case study Participants identified a list of priority research questions about food choice in these contexts.
Session 4: Setting priorities for analysis and further data collection and/or other studies References: TAFSSA survey questionnaires, DFC assessment methods repository	Participants identify and present a list of addressable research questions and additional data needs
Session 5: Developing analysis ideas for assessing food choice behaviors and drivers using TAFSSA food system assessment data References: TAFSSA survey questionnaires	Participants review descriptions of available data in study sites and prepare list of potential analyses
Session 6: Envisioning future publications and data needs for understanding DFC in South Asia	Participants present and discuss summaries, potential paper ideas and research activities

Abbreviations: DFC, Drivers of Food Choice; LMIC, low-income and middle-income country; TAFSSA, Transforming Agrifood Systems in South Asia.

On the first day of the workshop, the convening team opened with introductory presentations on TAFSSA’s framing of food choice and a presentation from the DFC team on using a science of food choice for understanding what, how, and why people eat the way they do [2]. Following the opening presentations, collaborators provided presentations on food systems contexts and examples of measurement of food choice. The first day concluded with presentations on progress toward creating a methods repository [34] for DFC and application of lessons learned over the course of the day with a hypothetical vignette.

Day 2 began with presentations on the nutrition situation in Bangladesh, India, and Nepal by representatives from in-country institutions working to improve nutrition and agriculture. These presentations provided context for facilitated group discussions. Four working groups were formed, 1 for each country (Bangladesh, India, and Nepal) and 1 online group. In-person participants were assigned to a country group based on the current location of the work or professional experience. The online participants worked on cross-country priorities (Bangladesh, India, and/or Nepal). All groups were tasked with identifying and prioritizing problems and discussing the needs for actionable evidence for food system transformation in each country. Groups were charged with considering what data were available to address identified priorities. All working groups had access to the household questionnaires of the TAFSSA local agrifood systems assessment survey [33] being administered across the 3 countries to 3 respondent types: male adult, female adult, and adolescent (either male or female).

The third and final day was structured to solicit input from participants on specific hypotheses and research questions that could be addressed to fill important evidence gaps using the TAFSSA data. Groups were formed to work on each of the identified research priorities, to develop and share the specific hypotheses and research questions. The meeting concluded with commitments by individual members and member organizations to participate in research, program, and outreach activities being coordinated by newly formed working groups for the different topics.

## Workshop Outputs: Priorities for DFC Research in South Asia

The workshop highlighted the importance of utilizing the science of food choice framework to guide data collection and understand what, how, and why people eat the way they do. The format of the workshop allowed for a comparison of needs from different country perspectives. Identification of research priorities reflected specific articulated needs of workshop participants for their country context that were not limited to the TAFSSA focus countries, as well as more general needs to advance the field. An additional output of this workshop was the identification of priorities for the development and testing of measures and tools to assess food choice behaviors (individual and household) and integrated measures and tools to evaluate the intersection of food choice behaviors and food environments.

The culmination of this process implemented on day 2 of the workshop was a set of 4 group presentations outlining priorities for each country context and crosscutting themes. The workshop convening team compiled outputs from day 2 and identified 3 important crosscutting themes that could be addressed using the TAFSSA agrifood systems assessment survey data. These were as follows: *1*) intergenerational differences in food choice behavior and preferences within households, *2*) adolescent food choice behaviors and their drivers, and *3*) food markets’ influence on food choice behaviors (Table 2). Specific research needs emphasized the importance of data on multigenerational and intrahousehold dynamics, food allocation, perspectives on food safety and healthy or unhealthy foods, adolescent food aspirations, exposure to food myths, and longitudinal data on how market availability impacts current and future choices of consumers and food vendors. Discussion included the importance of working with existing monitoring and evaluation systems in countries whenever possible to minimize need for expensive modification to existing systems.

**TABLE 2:** Immediate research priorities and research questions to answer with existing data to inform action.

Topic 1: Priorities for research on intrahousehold dynamics and individual behaviors		
• Understand intergenerational changes in drivers of food choice behavior to inform interventions that are relevant for multiple generations living together • Collect multigenerational data to understand the roles and influences of grandparents (in-laws) on food choice • Unpack intrahousehold allocation of foods (eating order, age/gender) • Understand how households organize themselves around food system-related tasks and the influencing factors—how might those change with seasonality and migration • Understand the role of food safety and hygiene on food choices • Examine leisure perceptions among different household members • Examine perceptions of what is “healthy” and “unhealthy” foods (nutrition literacy) • Examine self-perceptions about food choice (“I am a healthy eater.”) • Testing of interventions to increase intergenerational demand for foods produced in sustainable ways based on insights from food choice research	Subtopics	Research questions
	1. Intergenerational differences in food intake and food perceptions (grandparent vs. parent vs. adolescent)	• What are the intergenerational differences in food intake, if any? • How do the drivers of food choice for individual foods vary by generations represented within households?
	2. Gender differences in food intake and food perceptions (among parents and adolescents)	• What are the gender differences in food intake, if any? • How do the drivers of food choice for individual foods vary by gender?
	3. Consumption of foods outside the home	• How does the consumption of foods outside the home vary by gender and age?
	4. Food-related tasks in relation to food intake among different household members	• Who does what? How are intrahousehold tasks distributed across the production, acquisition, and consumption continuum?

Topic 2: Priorities for research on adolescent food choice behaviors and their drivers		
• Examine personal aspirations and food choices among adolescents, and how exposure to media and social values and relationships influence them • Conduct qualitative research on adolescent body image, dieting, and food neophobia—how do food ads influence these outcomes • Examine food myths and how these influence adolescent diets	Subtopics	Research questions
	5. Food advertisements, diet information, and food perceptions influence on adolescents’ diet	• How does exposure to food advertisements and diet information influence adolescent diet? Vary by life situation (marriage, in/out-of-school)? • How do food perceptions influence adolescent diet? Relative to exposure? Vary by life situation (marriage, in/out-of-school)?

On the final day of the workshop, participants committed to contributing to an analysis of existing and forthcoming data and further research addressing the identified priorities. Specific research questions and/or hypotheses for the 3 priorities identified and described in detail on day 2 were formulated and discussed (Table 2).

## Crosscutting Issues

Workshop participants also highlighted crosscutting issues that need to be addressed to successfully implement these research priorities. These issues were lack of high-quality data to guide decision making, lack of valid and reliable measures of DFC, and uncontrolled expansion of ultraprocessed food availability.

A major issue is a lack of high-quality data in part due to the lack of standardized, comprehensive tools and instruments to measure food choice and behaviors. Recent methodologic advancements to assess the food environments in LMICs [26,[35], [36], [37]] have emphasized the external food environment (i.e., availability, prices, vendors and product properties, marketing, and regulation) and, more recently, the personal food environment (i.e., accessibility, affordability, convenience, and desirability) [37]. Assessment of individual and household food choice behaviors and their drivers (e.g., values, habits, and priorities), however, has received less attention [2]. Workshop participants identified the dearth of valid and reliable instruments for the assessment of food choice behaviors and DFC as a constraint to policy and program development.

The role of ultraprocessed foods in the nutrition transition in South Asia is receiving more attention in the context of an increasing obesity and diet-related noncommunicable disease burden. Trends, patterns, and underlying drivers of processed food markets in this region, however, are not well understood. Questions about the nutritional value of affordable foods and beverages as well as increased access to affordable ultraprocessed foods and beverages were highlighted. Workshop participants emphasized the importance of understanding DFC that contribute to both food insecurity and undernutrition as well as overconsumption and overweight and obesity.

Workshop participants included researchers from universities and research centers (governmental and nongovernmental) in multiple South Asian countries and beyond. Although the CGIAR and its collaborators represent a broad network of researchers working on topics of nutrition, food and diets, policy makers, and program implementers were less represented in this workshop. Thus, the workshop was heavily focused on applied research experiences and evidence generated from research studies and less focused on policy engagement and large-scale implementation. Future work in this area would benefit from the inclusion of policy and program stakeholders.

## Discussion

The rate of transformation of food systems in South Asia is accelerating, presenting opportunities and challenges, particularly in urban areas. As people living in South Asia experience greater access to affordable foods and beverages, their food choices will have long-term effects on population health and well-being. Efforts to increase access to healthy foods while ultraprocessed food availability expands create new choice environments. Building a body of evidence on DFC and tools for monitoring and assessing food choice behaviors is essential for designing effective policies and programs to achieve consumption of sustainable healthy diets in South Asia. The type of multidisciplinary workshop presented in this article can be effective for identifying priorities, fostering collaborations, and generating multidisciplinary research agendas for the transformation of food, land, and water systems to achieve sustainable, food-secure, and climate-resilient communities.

## Author contributions

The authors’ responsibilities were as follows – CEB, EAF, SSK, PM: designed the workshop; CEB, SSK: were responsible for the design and writing the initial drafts of the manuscript; CEB, SSK, EAF, PM: were responsible for the final content of the manuscript; and all authors: have read and approved the manuscript.

## Funding

This research was funded by the CGIAR Trust Fund: www.cgiar.org/funders/.

## Conflicts of interest

The authors report no conflicts of interest.
